# Adoption of Artificial Intelligence–Based Precision Mental Health Technologies Among Psychology Trainees: Mixed Methods Cross-Sectional Survey Study

**DOI:** 10.2196/93893

**Published:** 2026-07-03

**Authors:** Sara Noheda, Eduar S Ramírez, Sara Rodriguez-Moreno, Carolina Martín-Azañedo, Ana Georgescu, Pablo Roca

**Affiliations:** 1 Precision Mind Lab (Premind), Faculty of Health Sciences, Universidad Villanueva, 2 Costa Brava Street, Madrid, Madrid, 28034, Spain, 34 917340413; 2 Universidad Francisco de Vitoria, Madrid, Spain; 3 Faculty of Biomedical and Health Sciences, Universidad Europea de Madrid, Madrid, Spain; 4 Medea Lab, Madrid, Spain

**Keywords:** artificial intelligence, AI, precision mental health, implementation, acceptability, intention to use, digital health, strategic planning

## Abstract

**Background:**

Despite the significant benefits of artificial intelligence (AI) in mental health, real-world implementation remains limited, making it essential to understand the factors that influence adoption.

**Objective:**

This study examined the acceptability and intention to use artificial intelligence–based precision mental health technologies (AI-PMHTs) and proposed an empirical, theory-guided model that integrates traditional technology acceptance predictors (eg, perceived usefulness, risk, and ease of use) with emerging psychological factors (eg, AI anxiety, personality, and conspiratorial thinking) that may inform future implementation research, strategic planning, and training program design.

**Methods:**

An online survey was distributed to a sample of 357 psychologists in training, including both undergraduate and master’s students. A mixed methods approach was used, combining quantitative measures (via psychometrically validated questionnaires) and qualitative data (through open-ended questions). Descriptive statistics and *t* tests were conducted to characterize the sample, and responses to the open-ended questions on facilitators and barriers were thematically analyzed. Partial least squares structural equation modeling was used to build the empirical model.

**Results:**

Participants showed moderate-to-high acceptance and intention to use AI-PMHTs, yet anxiety and perceived risk varied (with higher levels among women), and more frequent use was linked to more favorable acceptance profiles without reducing fear. Thematic analysis revealed that participants viewed AI tools as efficiency-enhancing but raised concerns about reliability, usability, overdependence, and access constraints. Partial least squares structural equation modeling supported a hierarchical adoption pathway in which dispositional and demographic factors shape AI-related fear and perceived risk, which then influence cognitive evaluations and attitudes, ultimately being associated with acceptance and intention to use AI-PMHTs. Predisposing variables (particularly resistance to change and conspiratorial thinking) were the strongest predictors of AI-related anxiety, with gender and extraversion showing smaller but meaningful effects. Fear acted as a key affective mediator, increasing perceived risk and indirectly weakening positive attitudes and perceived usefulness. Acceptance was the most influential downstream construct, directly predicting satisfaction, perceived usefulness, prior experience, and future intention to use, consistent with a reinforcing feedback loop in which early acceptance supports sustained engagement.

**Conclusions:**

Findings suggest a layered framework that may inform future implementation research and training program design, addressing (1) predisposing dispositional and emotional profiles; (2) precipitating fear and perceived risk via transparent regulation, explainable design, and policies that strengthen professional agency; and (3) maintenance through high-quality early experiences, usability, and sustained institutional support. This theory-guided model clarifies how psychological, contextual, and experiential factors jointly shape adoption and sustained use of AI-PMHTs among psychologists in training, informing targeted educational and implementation strategies for this population.

## Introduction

Mental health care systems worldwide are currently facing an urgent and multifaceted crisis. The global prevalence of mental disorders has increased significantly, with estimates suggesting that one in two individuals will develop a mental disorder at some point in their lives, and peak onset typically occurs around the age of 15 years [1]. Despite notable advancements in therapeutic approaches, the average effectiveness of psychological treatments has remained largely unchanged over the past 5 decades [2]. Only one-third of treated patients achieve full remission, while a significant proportion does not receive any treatment at all [3,4]. Structural barriers, such as long waiting lists, a shortage of mental health care professionals, and inadequate resources, continue to hinder treatment access [5]. Moreover, mental health care professionals themselves report feeling overwhelmed and highlight a lack of scalable solutions to meet growing demand [6].

The urgency of addressing long-standing limitations in mental health care has made the exploration of innovative technologies not only relevant but necessary, particularly as health care systems worldwide move toward strategic, evidence-based planning and the prioritization of digital health investments. A wide range of technological innovations has emerged in recent years as potential avenues for scalable and effective solutions [7]. These innovations include online and internet-based psychotherapies, wearable devices for digital phenotyping and real-time symptom monitoring, as well as immersive technologies such as virtual and augmented reality used for therapeutic purposes [6]. Digital mental health interventions have shown promise, especially among younger populations, offering enhanced anonymity, accessibility, and the potential for broad scalability [8,9]. However, among all these emerging technologies, artificial intelligence (AI) stands out as one of the most transformative innovations in mental health care, catalyzing a paradigm shift toward precision mental health [10]. Artificial intelligence–based precision mental health technologies (AI-PMHTs) refer to digital systems that integrate AI techniques (eg, machine learning, natural language processing, and predictive analytics) to support measurement-based care, ongoing monitoring, data-driven decision-making, and personalized interventions in mental health care (eg, clinical decision-support systems, predictive risk models, and similar tools) [11]. Due to their ability to analyze large-scale and multidimensional datasets, AI holds the potential to increase diagnostic accuracy and triage, personalize treatment plans, remotely monitor symptoms, and improve overall service efficiency [6,8]. By alleviating clinician workload and optimizing the use of limited resources, AI could play a pivotal role in expanding access to care and improving treatment outcomes [12]. In addition, certain AI-enabled interventions may help reduce barriers related to stigma, cost, and geographical inaccessibility, which continue to deter many individuals from seeking traditional mental health services [9].

Despite the significant benefits and potential of AI-PMHTs in mental health care, implementation in clinical practice remains limited, partly because the strategic planning required to guide these innovations is often inconsistently executed. Recent systematic reviews indicate that while AI applications such as chatbots, virtual assistants, and predictive analytics have shown promising results in experimental settings, their use in real-world clinical contexts remains rare and often confined to pilot projects or research initiatives [6]. For instance, fewer than 1% of individualized prediction models developed in mental health research have been evaluated for actual implementation in clinical settings [13], underscoring the persistent gap between innovation and application. A recent survey among mental health care professionals found that although over 70% acknowledge the potential of AI tools, fewer than 20% have actively integrated them into their practice [14].

Several factors have been identified as barriers to the widespread adoption of AI-PMHTs in mental health care. These include ethical and legal concerns, insufficient training in data-driven clinical decision-making, and challenges in integrating AI systems within diverse therapeutic frameworks (eg, humanistic, systemic, or psychodynamic models). Beyond structural and technical limitations, attitudinal and psychological variables (eg, trust, perceived usefulness, anxiety toward technology, and certain personality traits) also play a critical role in shaping professionals’ openness and willingness to use AI in clinical contexts [6,15]. Understanding how these psychological and contextual factors interact is essential to bridge the current gap between technological innovation and real-world implementation [16,17].

Over the past 2 decades, technology acceptance models have identified several core predictors influencing behavioral intention to use new technologies. One of the most widely applied frameworks is the Unified Theory of Acceptance and Use of Technology 2 (UTAUT-2) [18], which extends the original model by incorporating additional constructs relevant to voluntary technology use. Key predictors include performance expectancy (ie, perceived usefulness), effort expectancy (ie, perceived ease of use), social influence, facilitating conditions, hedonic motivation, price value, and habit. In addition to these core constructs, the UTAUT-2 framework recognizes that the strength and direction of these relationships can vary depending on individual and contextual moderators. Building on these foundations, further research has expanded this model to incorporate emotional and cognitive factors such as trust in technology, satisfaction with prior experiences, perceived enjoyment, perceived risk, and self-efficacy [6,19]. In mental health contexts, these predictors are particularly critical, given the sensitive and human-centered nature of psychological services.

However, although these traditional predictors explain a significant portion of technology adoption behavior, they do not capture the full range of psychological factors relevant to the acceptance of complex technologies such as AI [20]. Recent research has highlighted the importance of integrating emerging psychological variables into technology acceptance and implementation models. For instance, anxiety related to AI has been shown to negatively influence individuals’ willingness to adopt and engage with AI tools, particularly in sensitive contexts such as mental health [21]. Beyond anxiety, individual differences in personality and cognitive style have also been found to shape attitudes toward emerging technologies. Classical personality traits from the Big Five (particularly openness to experience, conscientiousness, and neuroticism) have been linked to differential patterns of technology acceptance and adaptability to innovation [22]. Individuals high in openness tend to display greater curiosity and tolerance for novelty, fostering positive attitudes toward emerging technologies, whereas those higher in neuroticism may exhibit stronger apprehension and risk perception. Moreover, dispositional resistance to change (ie, a stable tendency to avoid alterations in established routines) has been identified as a significant negative predictor of technology adoption, mediating the effects of perceived usefulness and trust [23]. Similarly, conspiratorial thinking and generalized distrust in institutions have been shown to amplify skepticism toward AI and data-driven systems, limiting perceived reliability and ethical acceptability [24]. Considering these insights, it becomes essential to incorporate these psychological predictors into technology acceptance frameworks to guide implementation science and translational research.

Only a limited number of empirical studies have examined the variables that influence attitudes and intention to use AI-PMHTs in mental health care. Surveys with mental health care professionals indicate a mixed outlook, characterized by recognition of potential benefits alongside concerns about ethics, therapeutic rapport, and limited technical preparation [6,8]. Existing research shows that clinicians generally perceive AI as useful for supporting tasks such as triage, symptom monitoring, or administrative processes, yet remain skeptical about its suitability for core therapeutic functions that rely on relational sensitivity and empathic attunement [8]. Psychiatrists and psychotherapists also report apprehension regarding issues of transparency, explainability, and potential erosion of clinical autonomy, which further contributes to ambivalent attitudes toward AI-based precision technologies [8,9]. Several studies highlight insufficient digital training and limited familiarity with measurement-based care and data-driven decision-making tools as major barriers to implementation, with many professionals feeling inadequately prepared to integrate these technologies into routine practice [25]. A consistent theme across this evidence is the educational and experiential gap required to critically evaluate and integrate AI-PMHTs in clinical contexts. This gap is best addressed early, during professional training. Accordingly, psychiatrists and psychologists in training constitute a key population to examine how knowledge, attitudes, and psychological factors shape AI acceptance. Recent work with future psychologists shows interest in AI but also highlights limited literacy and scarce curricular exposure, underscoring the need to strengthen digital and ethical competencies during training [6,12].

Understanding the factors that shape AI-PMHTs adoption is not only theoretically relevant but strategically necessary for effective implementation in real-world settings. However, current strategic planning in digital mental health often relies on intuition, vendor recommendations, or technology-driven decisions rather than comprehensive, evidence-based frameworks [15,16]. To our knowledge, few studies have extended existing technology acceptance and implementation frameworks by incorporating emerging psychological factors specific to AI adoption in mental health contexts. Therefore, this study aims to examine the acceptability and intention to use AI-PMHTs and propose an empirical, theory-guided model that integrates traditional technology acceptance predictors (eg, perceived usefulness, risk, ease of use, and social influence) with emerging psychological factors (eg, AI-related anxiety, personality traits, and conspiratorial thinking) that are critical for effective implementation and strategic planning. Consistent with this framework, we hypothesize that attitudes toward AI and AI use will differ as a function of gender, stage of training, and frequency of AI use. Furthermore, dispositional factors will influence intention to use AI-PMHTs through affective and cognitive processes, whereas AI acceptance will function as the principal mediator linking dispositional factors, attitudes, and facilitating conditions to AI experience and use and, ultimately, to behavioral intention to use AI-PMHTs.

## Methods

### Study Design

This study adopted a mixed methods design in the form of an online cross-sectional survey study, combining quantitative and qualitative approaches. Quantitative data were collected through psychometrically validated questionnaires to ensure reliability and construct validity. In addition, qualitative data were gathered through open-ended questions to complement and contextualize the quantitative findings.

### Participants

An online survey was distributed to a convenience sample of 357 psychologists in training, including undergraduates and master’s students. The initial sample comprised 358 students; however, 1 individual was excluded for failing the instructional manipulation check, which served as an attention check item [26]. Eligibility criteria were (1) being of legal age, (2) enrollment in undergraduate or postgraduate studies in psychology, (3) internet access, and (4) provision of signed informed consent. Regarding demographic characteristics, 84.3% (301/357) of participants identified as female (none identified as nonbinary; mean age: 20.71 years, SD 3.02 years), 59.4% (212/357) were single, and 31.3% (112/357) combined their studies with work. In terms of academic year, 26.9% (96/357) were first-year undergraduate students, 30% (107/357) were in their second year, 30% (107/357) were in their third or fourth year, and 13.2% (47/357) were enrolled in a master’s program.

### Procedure

The survey was distributed via the institutional mailing lists of the university participating in the project to recruit participants. Participants were informed that the survey was not a test and therefore did not contain right or wrong answers. At the beginning of the survey, participants were provided with a standardized definition of AI and AI-PMHTs, including examples, to ensure a shared understanding of the construct. They were also informed that the information provided would be treated in aggregate form, that responses would be pseudonymized, and that no sensitive personal data would be collected. Participation in the study was voluntary, and students received extra course credits as an academic incentive. Completion of the survey via Google Forms took approximately 15 minutes.

### Measures

#### Overview

We followed a mixed methods approach, combining quantitative measures (using psychometrically validated questionnaires) and qualitative measures (collected through open-ended questions).

#### Ad Hoc Survey

Specifically developed to collect demographic information (gender, age, and stage of training) and prior experience with AI (frequency of AI use, number of AI applications used, number of AI-enabled devices, and habitual use).

#### Acceptance and Use of Technology (UTAUT-2)

This instrument was used to assess variables involved in behavioral intention to use technology, adapted in this study to the context of AI. To capture additional affective and cognitive factors relevant to voluntary AI use, 5 complementary dimensions were incorporated from the extended version of UTAUT-2 [19]. Given the multiple predictors included in the original UTAUT-2 model and its subsequent extensions, we conducted an exploratory factor analysis to derive composite scores that provided a more parsimonious representation of higher-order constructs. The analysis yielded two broad factors: facilitating conditions (combining effort expectancy and facilitating conditions) and AI acceptance and predisposition (integrating social influence, performance expectancy, hedonic motivation, and trust). Responses were rated on a 7-point Likert scale ranging from 1 (strongly disagree) to 7 (strongly agree). Previous studies have reported adequate to excellent reliability for UTAUT-2 dimensions, with Cronbach α typically ranging from 0.76 to 0.96 and McDonald ω values above 0.80. In line with this evidence, the composite scores used in this study showed satisfactory internal consistency, with *ω*=0.84 for facilitating conditions and *ω*=0.87 for AI acceptance and predisposition.

#### Attitudes Toward Artificial Intelligence

A 12-item scale assessing general attitudes toward AI, integrating cognitive, affective, and behavioral components in evaluating perceptions of this technology. Participants responded using a 5-point Likert scale ranging from 1 (strongly disagree) to 5 (strongly agree), with higher scores indicating more positive attitudes toward AI [22]. The scale has shown high internal consistency with a Cronbach α of 0.89 and McDonald ω of 0.89, supporting its reliability.

#### Artificial Intelligence Anxiety Scale

A 21-item questionnaire designed to measure levels of anxiety and concerns regarding the use and presence of AI in various contexts. Responses were provided on a 7-point Likert scale ranging from 1 (strongly disagree) to 7 (strongly agree), where higher scores indicate greater AI-related anxiety [27]. The scale has demonstrated excellent reliability, with a global Cronbach α of 0.94 and ω of 0.94, indicating robust internal consistency.

#### Ten-Item Personality Inventory

A brief, 10-item measure derived from the Big Five model, allowing rapid assessment of personality traits (extraversion, agreeableness, conscientiousness, emotional stability, and openness to experience). Each item was rated on a 7-point Likert scale from 1 (strongly disagree) to 7 (strongly agree), with pairs of items representing each personality factor [28]. Although internal consistency for each of the 5 dimensions is modest (α≈0.40-0.68) due to the scale’s brevity, the Ten-Item Personality Inventory has demonstrated acceptable convergent validity and is widely used in large-scale research.

#### Resistance to Change Scale

An 11-item scale measuring individuals’ stable predisposition to resist change, encompassing dimensions such as routine seeking, emotional reaction, short-term focus, and cognitive rigidity. Items were rated on a 5-point Likert scale ranging from 1 (strongly disagree) to 5 (strongly agree), with higher scores reflecting greater resistance to change [23]. This scale has shown high reliability, with Cronbach α values of 0.90 and ω of 0.90, indicating strong internal consistency.

#### Conspiracy Mentality Questionnaire

A brief, 5-item scale used to assess the degree of conspiratorial thinking, understood as a generalized tendency to believe in conspiracies. Responses were made on an 11-point Likert-type scale from 0 (certainly not) to 10 (certainly), with higher scores indicating stronger endorsement of conspiratorial beliefs [29]. The Conspiracy Mentality Questionnaire has demonstrated good psychometric properties, with Cronbach α of 0.85 and ω of 0.86, confirming satisfactory reliability and validity.

#### Open-Ended Questions

Facilitators and barriers to the use of AI were assessed using the following two open-ended questions: (1) “What are the main benefits you have experienced when using AI?” and (2) “What are the main barriers you have encountered when using AI?”

### Data Analysis

Data analysis followed a structured, multistage procedure. As a preliminary step, data were preprocessed to ensure analytical robustness. The overall proportion of missing data was very low (0.3%); although Little's missing completely at random test was statistically significant (*χ*^2^_66_=385.00; *P*=.001), indicating that data were not missing completely at random. Missing values were imputed using predictive mean matching as implemented in the *mice* package of R (R Core Team) [29], generating m=5 imputed datasets. Given the negligible proportion of missing data, the between-imputation variance was expected to be trivial; therefore, analyses were conducted on a single completed dataset (the first of the 5 imputed datasets), consistent with recommendations for low-missingness scenarios. This approach was verified by confirming the consistency of results across all 5 imputed datasets. All numeric variables were subsequently standardized into *z* scores to facilitate comparability across predictors and interpretation of model coefficients. Data preparation and management were conducted using the *dplyr* package of R [30].

Following preprocessing, the analyses were conducted in four sequential stages: (1) descriptive statistics and group comparisons were conducted using independent-samples *t* tests to characterize the sample and examine differences as a function of gender, stage of training (first-year undergraduate vs master’s students), and frequency of AI use (high vs low use); effect sizes were estimated using Hedges g and interpreted according to conventional benchmarks (small≈0.20, medium≈0.50, and large≈0.80); (2) responses to facilitators and barriers open-ended questions were analyzed using thematic analysis following the approach proposed by Braun and Clarke [31], with 2 independent evaluators involved in the coding process and discrepancies discussed with a third evaluator to refine and agree on the final themes; (3) in line with methodological recommendations for explanatory-predictive research and theory-driven model development [32,33], a series of sequential, thematically oriented multiple regression analyses were conducted to identify the most relevant predictors and reduce model complexity prior to structural modeling. These analyses were designed as a predictor-screening strategy, and their results should therefore be interpreted as a data reduction step, not as a direct test of the theoretical model. All regression models were estimated using the *lm*() function from the base *stats* package of R [34]; and (4) partial least squares structural equation modeling (PLS-SEM) was estimated using the *seminr* package of R [34] to integrate the predictors identified in the sequential multiple regression analyses into a unified explanatory framework. A fully specified baseline structural model was initially defined, including all theoretically plausible relationships among the retained predictors. This model was subsequently refined through a systematic pruning process, whereby nonsignificant structural paths were removed, resulting in a more parsimonious final model that preserved theoretical coherence while retaining only empirically supported relationships. Model evaluation followed established PLS-SEM guidelines [34]. Internal consistency reliability was assessed using composite reliability, with all constructs exceeding the recommended threshold of 0.70. Convergent validity was supported by average variance extracted values above 0.50, in accordance with the criteria proposed by Fornell and Larcker [35]. Discriminant validity was examined using the heterotrait-monotrait ratio [36], with all values remaining below the conservative cutoff of 0.85. Multicollinearity was evaluated through variance inflation factors, all of which were well below the recommended threshold of 3, indicating the absence of problematic collinearity among predictors [34]. Additionally, effect size indices (f²) [37] were computed to assess the substantive contribution of each exogenous construct to its corresponding endogenous variable; values of 0.02, 0.15, and 0.35 were interpreted as small, medium, and large effects, respectively [34]. Statistical significance of direct and indirect effects was assessed using a bootstrapping procedure with 5000 resamples, from which 95% CIs were derived for all parameter estimates, ensuring robust inference.

### Ethical Considerations

This study was approved by the Ethics Committee of Universidad Villanueva (VN-2024‐26). All procedures performed in this study involving human participants were conducted in accordance with the ethical standards of the institutional research committee. Electronic informed consent was obtained from all participants before accessing the survey. Participants were informed of the voluntary nature of the study and their right to withdraw at any time without academic penalty. Extra course credits were provided as an academic incentive for participation. All analyses were conducted on pseudonymized datasets, and findings were reported exclusively in aggregate form to prevent identification of individual participants.

## Results

### Descriptive Statistics and Group Differences

Descriptive statistics and group differences are reported in Multimedia Appendix 1. Overall, participants reported moderate-to-high levels of positive attitudes toward AI, facilitating conditions, and acceptance and predisposition toward AI, as well as generally high perceived usefulness, satisfaction, and future intention to use. In contrast, perceived risk and AI-related anxiety showed greater variability across the sample (Table S1 in Multimedia Appendix 1). Regarding gender differences, women reported higher levels of AI-related anxiety than men (*t*_79.68_=−3.86; *P*<.001; *g*=−0.55), whereas men reported slightly higher facilitating conditions (*t*_83.99_=2.03; *P*=.04; *g*=0.27). The magnitude of these differences was moderate for AI-related anxiety and small for facilitating conditions. No statistically significant gender differences were observed for perceived risk, positive attitudes toward AI, acceptance and predisposition, prior experience, usefulness, satisfaction, frequency of use, or future intention to use (Table S2 in Multimedia Appendix 1). Differences by stage of training indicated that master’s students reported more positive attitudes toward AI (*t*_99.33_=−2.20; *P*=.03; *g*=−0.38), whereas first-year undergraduate students reported higher facilitating conditions (*t*_86.69_=3.57; *P*=.001; *g*=0.67). Effect sizes ranged from small-to-moderate for positive attitudes toward AI, and from moderate-to-large for facilitating conditions. No additional differences were observed between training stages for the remaining variables (Table S3 in Multimedia Appendix 1). Finally, differences emerged as a function of frequency of AI use: participants reporting high frequency of use showed more positive attitudes toward AI (*t*_201.04_=−3.97; *P*<.001; *g*=−0.47), greater facilitating conditions (*t*_227.36_=−5.28; *P*<.001; *g*=−0.60), higher acceptance and predisposition (*t*_198.46_=−5.56; *P*<.001; *g*=−0.66), greater prior experience (*t*_186.30_=−9.35; *P*<.001; *g*=−1.11), higher perceived usefulness (*t*_297.25_=−9.97; *P*<.001; *g*=−1.02), greater satisfaction (*t*_256.79_=−6.62; *P*<.001; *g*=−0.74), and stronger future intention to use (*t*_211.73_=−7.62; *P*<.001; *g*=−0.88), compared with low-frequency users. No differences were observed between frequency-of-use groups for perceived risk or AI-related anxiety, suggesting that more frequent engagement with AI tools may selectively strengthen cognitive and behavioral dimensions of adoption without reducing affective responses such as fear or perceived threat. Effect sizes ranged from small to moderate, with the largest effects observed for prior experience and perceived usefulness (Table S4 in Multimedia Appendix 1).

### Thematic Analysis of Facilitators and Barriers to Adopting AI-PMHTs

Table 1 summarizes the facilitators and barriers identified through thematic analysis, along with the percentage of responses in which each theme was identified and illustrative verbatim excerpts. Participants described AI as a supportive resource that enhances efficiency in work activities, particularly by facilitating access to information. They also highlighted its value in helping them clarify complex concepts, structure assignments, and plan job tasks more effectively. In addition, several responses emphasized AI’s constant availability and nonjudgmental nature, which participants perceived as enabling repeated questioning and personalized assistance. Taken together, these themes portray AI as a useful tool that promotes learning, organization, and productivity.

**Table 1.** Thematic analysis of facilitators and barriers to the adoption of artificial intelligence–based precision mental health technologies. Percentages indicate the proportion of responses in which each theme was identified (facilitators: n=346; barriers: n=345). Because responses could be coded into more than 1 theme, percentages do not sum to 100.

Theme	Description	Percentage of responses	Example verbatim
Facilitators			
Rapid access to information	AI^a^ provides immediate access to information and answers, reducing the need for extensive searching across multiple sources.	27.2%	“I can get information instantly whenever I need it.” (ID001)
Support for learning and understanding	AI helps clarify complex concepts, offers alternative explanations, and adapts content to students’ comprehension levels.	47.7%	“It explains things in different ways until I understand them.” (ID007)
Efficiency and time savings	AI facilitates faster completion of academic tasks, such as summarizing texts, resolving doubts, or preparing assignments.	24.3%	“It saves a lot of time when doing coursework.” (ID079)
Organization and structuring of work	AI assists in organizing ideas, structuring texts, planning tasks, and preparing materials.	11.6%	“It helps me organize my ideas and structure my assignments.” (ID008)
Continuous availability and personalized support	AI is perceived as constantly available and nonjudgmental, allowing repeated questions and personalized learning support.	4%	“I can ask as many times as I want without feeling judged.” (ID099)
Barriers			
Lack of reliability and inaccuracies	Concerns about incorrect, imprecise, outdated, or misleading information generated by AI.	31.9%	“Sometimes it gives wrong answers.” (ID326)
Difficulties in use or interaction	Problems related to formulating effective prompts, being understood by the system, or receiving relevant responses.	17.7%	“Sometimes it doesn’t understand what you’re asking.” (ID344)
Risk of overdependence	Concerns that frequent AI use may reduce effort, critical thinking, creativity, or autonomous learning.	11.3%	“You might stop thinking for yourself.” (ID113)
Limitations in specific tasks	AI performs inconsistently in certain tasks, particularly numerical exercises, complex calculations, or highly specific academic questions.	9.3%	“It doesn’t work well for some math problems.” (ID103)
Access and contextual constraints	Barriers related to usage limits, paid features, technical restrictions, or institutional constraints regarding AI use.	8.4%	“Some features require a paid premium version.” (ID066)

a AI: artificial intelligence.

Participants expressed concerns regarding the reliability and accuracy of AI-generated output, noting instances of incorrect or misleading information. They also reported difficulties interacting with the systems, particularly when formulating prompts or obtaining relevant responses. Further responses highlighted apprehension about overdependence on AI tools, with worries that heavy use could reduce critical thinking or independent learning efforts. Participants additionally mentioned limitations when AI was applied to highly specific tasks, numerical exercises, or complex job problems, as well as practical access barriers such as usage restrictions or subscription requirements. Notably, a subset of participants indicated that they did not perceive any significant obstacles associated with AI use.

### Empirical Model of Acceptability and Intention to Use AI-PMHTs

The PLS-SEM model was estimated using the significant predictors previously identified in the sequential regressions (refer to Tables S5-S11 in Multimedia Appendix 1), enabling a unified examination of traditional technology acceptance predictors (eg, perceived usefulness, risk, ease of use, and social influence) and emerging psychological factors (eg, AI-related anxiety, personality traits, and conspiratorial thinking). All latent constructs met recommended psychometric standards: composite reliability values exceeded 0.70 and average variance extracted values were above 0.50, supporting convergent validity. Discriminant validity was confirmed through both the Fornell-Larcker criterion and heterotrait-monotrait ratio values below 0.85, while all variance inflation factor values remained below 2, indicating no problematic multicollinearity across the measurement and structural models. The structural model demonstrated substantial predictive power, with future intention to use showing the highest explained variance (*R*^²^=0.61), followed by AI acceptance and predisposition (*R*^²^=0.50) and usage frequency (*R*^²^=0.43).

**Figure 1.**
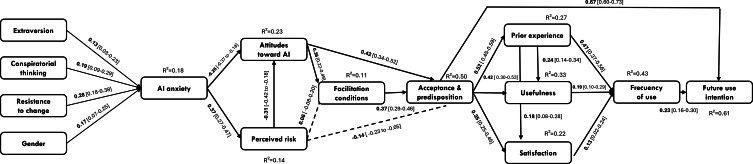
Partial least squares structural equation modeling of artificial intelligence (AI)-based precision mental health technologies adoption. The values displayed on the arrows represent standardized path coefficients (*β*). Bracketed values indicate 95% CIs obtained through bootstrapping (5000 resamples). Solid lines represent positive relationships, whereas dashed lines indicate negative relationships. Values displayed above each construct correspond to explained variance (*R*²).

Structural results are presented following the left-to-right ordering of the model depicted in Figure 1, focusing on the estimated direct effects among constructs. In the initial segment of the model, demographic and dispositional predictors showed significant direct associations with emotional predictors: resistance to change (*β*=.28, 95% CI 0.18-0.38; *f*^²^=0.09), conspiratorial thinking (*β*=.19, 95% CI 0.09-0.29; *f*^²^=0.04), extraversion (*β*=.13, 95% CI 0.03-0.23; *f*^²^=0.02), and gender (*β*=.16, 95% CI 0.07-0.25; *f*^²^=0.03) were positively related to AI anxiety, accounting for a substantial proportion of variance in this construct (*R*^²^=0.18).

AI anxiety exhibited significant direct effects on the evaluative and cognitive components that follow in the model, increasing perceived risk (*β*=0.37, 95% CI 0.27-0.47; *f*^²^=0.16) and reducing positive attitudes toward AI (*β*=−.28, 95% CI −0.37 to −0.18; *f*^²^=0.09). Perceived risk also showed a negative association with positive attitudes (*β*=−0.30, 95% CI −0.42 to −0.18; *f^²^*=0.10). Together, these paths accounted for meaningful variance in perceived risk and positive attitudes (*R*^²^=0.23). In the next segment, positive attitudes toward AI showed positive direct effects on facilitating conditions (*β*=.36, 95% CI 0.22-0.49; *f*^²^=0.12), as well as a positive direct effect on acceptance and predisposition toward AI (*β*=.42, 95% CI 0.34-0.52; *f*^²^=0.27). Facilitating conditions also exerted a significant direct effect on acceptance and predisposition (*β*=.37, 95% CI 0.29-0.46; *f^²^*=0.25), resulting in high explained variance for this construct (*R*^²^=0.50).

Downstream, acceptance and predisposition toward AI displayed strong direct associations with behavioral outcomes. Higher acceptance directly predicted prior experience with AI (*β*=.52, 95% CI 0.45-0.59; *f^²^*=0.37), perceived utility (*β*=.42, 95% CI 0.29-0.53; *f*^²^=0.19), and satisfaction (*β*=.35, 95% CI 0.25-0.45; *f*^²^=0.12). Prior experience, in turn, showed a strong direct effect on usage frequency (*β*=.47, 95% CI 0.37-0.56; *f*^²^=0.30), alongside additional direct contributions from satisfaction (*β*=.13, 95% CI 0.02-0.24; *f^²^*=0.02) and perceived utility (*β*=.19, 95% CI 0.10-0.29; *f^²^*=0.04). Finally, future intention to use AI-PMHTs was primarily explained by a strong direct effect of acceptance and predisposition toward AI (*β*=.67, 95% CI 0.60-0.73; *f*^²^=1). Usage frequency also showed a significant direct association with future intention to use (*β*=.23, 95% CI 0.16-0.31; *f^²^*=0.12). Collectively, these direct effects accounted for a substantial proportion of variance in future intention to use (*R^²^*=0.61), as illustrated in Figure 1.

Beyond the direct paths, the PLS-SEM model revealed a systematic pattern of statistically significant indirect effects that further clarifies how earlier model components are linked to downstream outcomes through mediational processes (Table S12 in Multimedia Appendix 1). At the initial stage of the model, dispositional and demographic variables exhibited significant indirect effects on multiple downstream constructs through their associations with AI-related anxiety. Extraversion showed negative indirect effects on positive attitudes (*β*=−.05, 95% CI −0.09 to −0.01), acceptance and predisposition toward AI (*β*=−.03, 95% CI −0.06 to −0.01), and future intention to use (*β*=−.03, 95% CI −0.05 to −0.01). Similarly, conspiratorial thinking exerted negative indirect effects on positive attitudes (*β*=−.08, 95% CI −0.12 to −0.03), acceptance and predisposition (*β*=−.05, 95% CI −0.08 to −0.02), and future intention to use (*β*=−.04, 95% CI −0.06 to −0.02). Resistance to change showed the strongest pattern of indirect associations among dispositional variables, including effects on attitudes (*β*=−.11, 95% CI −0.16 to −0.06), acceptance and predisposition (*β*=−.07, 95% CI −0.11 to −0.04), and future intention to use (*β*=−.06, 95% CI −0.08 to −0.03). Gender also displayed small but significant indirect effects on acceptance and predisposition (*β*=−.04, 95% CI −0.07 to −0.02) and future intention to use (*β*=−.03, 95% CI −0.06 to −0.01).

Within the subsequent segment of the model, AI-related anxiety showed robust indirect effects on downstream constructs via perceived risk and attitudes. AI anxiety was indirectly associated with acceptance and predisposition toward AI (*β*=−.26, 95% CI −0.33 to −0.19), facilitating conditions (*β*=−.12, 95% CI −0.18 to −0.06), and future intention to use (*β*=−.20, 95% CI −0.26 to −0.15). Perceived risk also demonstrated significant indirect effects on acceptance and predisposition (*β*=−.15, 95% CI −0.23 to −0.07) and future intention to use (*β*=−.22, 95% CI −0.31 to −0.14), reflecting its propagation through attitudinal and acceptance-related pathways.

Further along the model, attitudes toward AI exhibited positive indirect effects on acceptance and predisposition (*β*=.13, 95% CI 0.08 to 0.20) and on all experiential variables. Specifically, attitudes showed indirect associations with prior experience (*β*=.29, 95% CI 0.22-0.37), satisfaction (*β*=.25, 95% CI 0.18-0.32), perceived utility (*β*=.30, 95% CI 0.23-0.38), usage frequency (*β*=.23, 95% CI 0.17-0.29), and future intention to use (*β*=.43, 95% CI 0.34-0.51). Facilitating conditions similarly displayed positive indirect effects on experiential outcomes, including prior experience (*β*=.19, 95% CI 0.14-0.25), satisfaction (*β*=.17, 95% CI 0.12-0.22), perceived utility (*β*=.20, 95% CI 0.15-0.26), usage frequency (*β*=.15, 95% CI 0.11-0.20), and future intention to use (*β*=.29, 95% CI 0.21-0.35).

Finally, acceptance and predisposition toward AI showed significant indirect effects on future intention to use through experiential variables. Acceptance was indirectly associated with usage frequency (*β*=.41, 95% CI 0.35-0.47) and future intention to use (*β*=.10, 95% CI 0.06-0.13) via prior experience and subsequent engagement. Prior experience also exerted an indirect effect on future intention to use through usage frequency (*β*=.13, 95% CI 0.08-0.18). Smaller but significant indirect effects were observed for satisfaction (*β*=.03, 95% CI 0.01-0.06) and perceived utility (*β*=.05, 95% CI 0.02-0.07) on future intention to use, operating through frequency of use.

## Discussion

### Principal Findings

Building on the study’s overarching aim to propose an empirical, theory-guided model of AI-PMHTs adoption in mental health care, this research examined the acceptability and intention to use AI-PMHTs by integrating traditional technology acceptance predictors (eg, perceived usefulness, risk, ease of use, and social influence) with emerging psychological factors (eg, AI-related anxiety, personality traits, and conspiratorial thinking) that are critical for effective implementation and strategic planning.

Overall, participants reported generally positive evaluations of AI and moderate-to-high levels of acceptance and future intention to use. At the same time, perceived risk and AI-related anxiety showed greater variability, suggesting that emotional responses to AI are less homogeneous and may constitute early sources of resistance in specific subgroups. These descriptive findings indicate that openness to AI coexists with underlying ambivalence, reinforcing the need to examine both affective and cognitive dimensions of adoption [8,9]. Regarding demographic differences, women reported higher levels of AI-related anxiety, whereas men perceived slightly more favorable facilitating conditions. Prior studies indicate that women, on average, report higher levels of technology-related anxiety and lower perceived readiness when engaging with advanced digital systems, whereas men tend to perceive more favorable facilitating conditions and greater technological confidence [38,39]. Importantly, these differences have been attributed primarily to sociocultural and contextual factors (eg, differential exposure to technology, gendered socialization, and stereotypes) rather than to differences in objective ability [40]. The replication of this gender pattern within the context of AI-PMHTs suggests that emerging digital health innovations may inadvertently reproduce longstanding science, technology, engineering, arts, and mathematics–related disparities unless gender-sensitive training, communication, and implementation strategies are explicitly integrated. However, the sample showed a strong gender imbalance (although this may reflect the demographic distribution of psychology programs), which may limit the interpretation of gender differences observed in the study.

Differences by stage of training were similarly nuanced: master’s students exhibited more positive attitudes toward AI, while earlier-stage students perceived stronger facilitating conditions. More pronounced differences emerged as a function of frequency of AI use: participants who reported more frequent use consistently showed more favorable acceptance profiles and stronger future intention to use. These patterns underscore the central role of experience in shaping AI acceptance, suggesting that engagement with AI tools reinforces positive evaluations across cognitive, affective, and behavioral domains [18,19]. Notably, fear and perceived risk did not differ as a function of use frequency, indicating that exposure to AI tools selectively strengthens the cognitive and behavioral components of adoption without reducing its affective component. This pattern is consistent with models of technology-related stress that distinguish between competence-based anxiety, which tends to diminish with practice, and identity-based or displacement anxiety, which may persist regardless of skill acquisition [41]. In the context of mental health care professionals, AI-related fear may be partially rooted in broader concerns about professional identity, therapeutic uniqueness, and societal narratives around automation (concerns that direct exposure to a specific tool is unlikely to resolve on its own). From an implementation perspective, this finding suggests the importance of addressing the affective dimensions of AI adoption through targeted educational and institutional strategies, rather than assuming that increased use alone will normalize emotional responses to AI [40].

Complementing the quantitative findings, the thematic analysis of facilitators and barriers helped contextualize several of the predictors included in the model. Frequently reported facilitators, such as efficiency, rapid access to information, and support for learning, are consistent with higher perceived usefulness and more favorable attitudes toward AI-PMHTs [18]. In addition, participants’ descriptions of AI as a resource that makes academic tasks easier to manage are compatible with the role of facilitating conditions in promoting acceptance and future use. In contrast, barriers such as reliability concerns, interaction difficulties, and overdependence help explain the continued relevance of perceived risk and AI-related anxiety in shaping attitudes and readiness for adoption [21]. These findings are also in line with previous research showing that mental health care professionals tend to recognize the practical utility of AI while simultaneously expressing concerns about trust, reliability, and appropriate use boundaries [7,8]. Taken together, the qualitative findings reinforce the interpretation of the quantitative model by showing how participants’ practical experiences with AI map onto the cognitive and affective processes underlying acceptance and intention to use.

In line with the study objectives and hypotheses, the PLS-SEM results revealed a hierarchical configuration in which stable dispositional and demographic characteristics were associated with emotional responses (eg, fear and perceived risk), which subsequently were associated with cognitive evaluations and attitudes toward AI-PMHTs, ultimately affecting acceptance and behavioral intention to use. This pattern supports the hypothesized assumption that adoption is not driven solely by traditional technology acceptance factors [42] but emerges from the interaction between classical predictors and emerging psychological variables that are critical for AI adoption [22].

Focusing first on predisposing factors, the results indicate that dispositional and emotional predictors play a critical role in shaping initial responses to AI-PMHTs. Personality traits and belief systems, particularly resistance to change and conspiratorial thinking [22,23], emerged as the strongest predictors of AI-related anxiety, while gender and extraversion showed more moderate but meaningful associations. These findings suggest that the earliest barriers to AI adoption arise before professionals engage with specific tools or workflows, operating instead at a psychological level characterized by anticipatory threat, distrust, and emotional resistance [21]. This pattern supports models of technology adoption that emphasize the primacy of affective predispositions in shaping downstream cognitive and behavioral processes [42,43]. Individuals characterized by higher rigidity may be more likely to perceive AI as opaque, uncontrollable, or professionally threatening, thereby amplifying fear responses that constrain openness to adoption. In contrast, more extraverted profiles appear to approach AI with greater curiosity and reduced emotional threat, facilitating engagement with subsequent stages of the adoption process [22]. From a digital health strategic planning perspective, these findings suggest the importance of considering dispositional heterogeneity within the mental health workforce. Future implementation studies and training programs could examine the value of avoiding assumptions of uniform readiness for AI adoption and of incorporating early-stage strategies aimed at identifying professional profiles that may be particularly vulnerable to fear-driven resistance. Differentiated onboarding pathways, combining transparent communication, acknowledgment of professional concerns, and institutional narratives that frame AI as a supportive rather than substitutive technology, may help prevent dispositional fear from becoming a structural barrier to later acceptance and use.

Beyond predisposing characteristics, the model identifies a set of precipitating factors that determine whether initial emotional responses evolve into acceptance or rejection. AI-related fear emerged as a central affective mediator, amplifying perceived risk and indirectly undermining positive attitudes toward AI. Perceived risk, in turn, exerted a dual influence by intensifying negative evaluations while indirectly undermining perceived usefulness through its effects on attitudes and acceptance. Together, these findings suggest that affective and cognitive processes act in a complementary manner, shaping the transition from predisposition to behavioral readiness [44]. This pattern aligns with previous research identifying fear and uncertainty as key barriers to adoption in high-stakes domains such as mental health care [21]. Importantly, the present results suggest that fear does not operate in isolation but is translated into acceptance outcomes through cognitive appraisals, including attitudes, risk perception, and beliefs about control and utility [18]. This highlights a critical window for intervention, where organizational, regulatory, and technological factors can reshape perceptions before resistance becomes entrenched. From an implementation standpoint, these findings may underscore the need for coordinated strategies that extend beyond education alone: clear regulatory frameworks, transparent certification processes, and validated clinical guidelines are essential to reduce perceived risk and enhance trust in AI-PMHTs.

Finally, the model reveals a set of factors responsible for the maintenance and future use (ie, behavioral predictors) of AI-PMHTs. Acceptance emerged as the most influential variable in the model, showing strong associations with satisfaction, perceived usefulness, prior experience, and future intention to use. This configuration suggests a reinforcing feedback loop in which early acceptance strengthens perceived control and value, generating positive experiences that sustain continued use over time. In this sense, acceptance functions not merely as an outcome, but as a central driver of long-term engagement. Recent work on human-AI collaboration further supports this interpretation, showing that sustained and effective use depends not only on technological performance but also on users’ ability to build accurate mental models of the system and engage in collaborative interaction with the tool [44]. In our study, acceptance reflects dispositional openness and a voluntary predisposition to engage with AI technologies. In contexts of nonmandatory use, such as the voluntary use of AI-PMHTs, this predisposition may facilitate exploratory behavior and voluntary exposure to AI tools, which, in turn, may contribute to accumulated experience. In this sense, the proposed path from acceptance to prior experience is not intended to imply a strict temporal sequence, as the cross-sectional nature of the data precludes causal or temporal inferences. Rather, it reflects a theoretically informed association whereby favorable predispositions toward AI may facilitate engagement behaviors that are reflected in greater exposure. These findings may point to the importance of the quality of early user experiences, as frustration, misunderstanding, or misalignment between user expectations and system behavior can undermine confidence and motivation, even when the underlying technology is robust [16]. From a future implementation research perspective, these results suggest that studies conducted in applied settings (eg, hospitals, community mental health services, and private practices) could examine whether structured pilot programs that prioritize usability, transparency, and professional support facilitate responsible AI-PMHT adoption. Future studies should also examine whether protected environments for initial use (eg, supported by multidisciplinary supervision, technical assistance, and clear clinical protocols) are associated with more positive user experiences and sustained engagement over time.

These findings align closely with contemporary implementation frameworks [16], which emphasize multilevel interventions across attitudinal, technological, statistical, and contextual domains. In addition, recent systematic reviews highlight that successful AI integration in health care requires deliberate planning around workforce competencies, including ethical reasoning, interpretative skills, and practical decision-making capabilities [15]. Taken together, the findings suggest a layered, theory-informed framework that may guide future research on digital mental health training and implementation planning that mirrors the structure of the proposed model. First, future training and implementation research could examine strategies that address predisposing factors by recognizing that dispositional and emotional profiles shape initial readiness and may require differentiated engagement strategies. Second, future intervention studies could test whether targeting precipitating factors by reducing fear and perceived risk through transparent regulation, explainable AI design, and organizational policies that enhance professional agency and trust. Third, future work could evaluate whether maintaining factors are supported by high-quality early user experiences, usability-focused system design, and sustained institutional backing. By articulating this sequence within a theory-guided empirical model, the present study advances understanding of how psychological, contextual, and experiential processes jointly shape the adoption and sustained use of AI-PMHTs. Importantly, the distinction between predisposing, precipitating, and maintaining factors should be understood as a theory-informed conceptual framework rather than a confirmed temporal or causal sequence and should be further examined in future longitudinal research.

Among the main strengths of this study is the theory-driven model, which combines predisposing, precipitating, and maintaining variables (including traditional technology acceptance predictors and emerging psychological factors) within an integrative empirical framework. Each predictor was selected based on an extensive review of prior scientific literature, ensuring conceptual relevance and theoretical grounding. The hierarchical organization of the model reflects established psychological and technology acceptance theories while extending them to the context of AI-PMHTs. Furthermore, the use of advanced data-analytic techniques (eg, PLS-SEM) enabled robust estimation of complex mediating relationships and strong predictive accuracy.

Several limitations should also be acknowledged. The cross-sectional design precludes causal inference, and the reliance on self-report measures may introduce biases related to social desirability or the novelty of AI technologies. Accordingly, all relationships identified in this study should be interpreted as associations rather than causal effects. Furthermore, although the sample was appropriate for exploratory and theory-building purposes, its academic context limits generalizability to applied clinical settings. The sample primarily consisted of psychologists in training who received participation incentives, and therefore, the findings should be interpreted as reflecting attitudes toward AI-PMHTs among future professionals rather than practicing clinicians or real-world mental health care systems. Furthermore, while some constructs (eg, prior experience and AI-related anxiety) refer to general-purpose AI systems, the key outcome variables (eg, acceptance and intention to use) were specifically framed in relation to professional applications within mental health contexts (eg, measurement-based care and data-informed decision-making). Regarding the analytical approach, the supplementary blockwise regressions and the final PLS-SEM model should be understood as answering different analytical questions. The regression analyses were used as a predictor-screening and data-reduction step, whereas the PLS-SEM model reflects a theory-specified structural representation of the retained constructs. Accordingly, the counterintuitive coefficients observed in some supplementary regression models should be interpreted cautiously and should not be treated as confirmatory evidence. Similarly, the final PLS-SEM model should not be interpreted as definitively resolving those coefficients, but rather as providing a theoretically coherent framework for examining the broader pattern of associations among constructs. Regarding the use of the Ten-Item Personality Inventory, while suitable for brief personality assessments, it is associated with relatively low internal consistency, which may introduce measurement error and affect SEM estimates. For all the above reasons, this study does not aim to demonstrate the real-world clinical implementation of AI-PMHTs, but rather to propose an exploratory, theory-guided model of how psychological and attitudinal factors may influence readiness and predisposition among future mental health care professionals.

Future research should address these limitations through longitudinal designs, multimethod assessment strategies, and samples drawn from diverse health care environments. Research should also explore AI adoption within real clinical workflows, including hospitals, community mental health services, and private practices, to capture the influence of organizational culture, workload, and regulatory constraints. Finally, policy-oriented research is essential to identify governance frameworks, accreditation standards, and reimbursement strategies that support ethical, effective, and scalable AI implementation in mental health care.

### Conclusions

In summary, the findings support a layered approach to digital mental health strategic planning that mirrors the structure of the proposed model. First, strategic initiatives should address predisposing factors by recognizing that dispositional and emotional profiles influence initial readiness and may require differentiated engagement strategies. Second, implementation strategies may target precipitating factors by reducing fear and perceived risk through transparent regulation, explainable AI design, and organizational policies that enhance professional agency and trust. Third, maintaining factors may be supported through high-quality early experiences, usability-focused system design, and sustained institutional backing. By articulating this sequence within a theory-guided empirical model, this study contributes to the emerging literature on AI-PMHTs adoption in psychology and mental health care. Importantly, these findings should be interpreted as reflecting the attitudes and readiness of future professionals in training rather than as a direct explanation of AI implementation in real-world health care systems. However, these insights have practical implications for the design of training programs, implementation strategies, and institutional policies aimed at facilitating responsible and effective AI integration in mental health settings. Future research should examine how these dynamics evolve across professional stages and clinical contexts, as well as how targeted interventions can optimize adoption pathways over time.

## Supplementary material

10.2196/93893 Multimedia Appendix 1 Methods, results, and tables.

## Abbreviations

AI: artificial intelligence
AI-PMHT: artificial intelligence–based precision mental health technology
PLS-SEM: partial least squares structural equation modeling
UTAUT-2: Unified Theory of Acceptance and Use of Technology 2
