# Supplementary material for: Adoption of Artificial Intelligence–Based Precision Mental Health Technologies Among Psychology Trainees: Mixed Methods Cross-Sectional Survey Study
Source: J Med Internet Res. 2026 Jul 3;28:e93893. doi: 10.2196/93893 (PMC13331070; doi:10.2196/93893)
Supplement: Multimedia Appendix 1 [file jmir-v28-e93893-s001.pdf]

1 Descriptive statistics and group differences

Descriptive statistics and group differences for all study variables are reported in Multimedia Appendix 1 (Tables S1–S4). Overall, participants reported moderate-to-high levels of positive attitudes toward AI, facilitating conditions, and acceptance and predisposition toward AI. Perceived usefulness, satisfaction, and future intention to use also showed relatively high mean values. In contrast, perceived risk and AI-related anxiety exhibited greater variability across the sample (Table S1).

Table S1: Descriptive Statistics of Measured Variables (*N* = 357)

Variable	*Min*	*Max*	*M*	*SD*	ω
AI Anxiety	24.00	145.00	91.63	20.92	0.93
Perceived Risk	1.00	7.00	4.26	0.97	0.60
Positive Attitudes	17.00	56.00	39.34	6.61	0.85
Facilitating Conditions	1.38	7.00	5.10	0.81	0.84
Acceptance & Predisposition	2.08	6.75	4.55	0.76	0.87
Prior Experience	0.33	5.17	2.52	0.72	0.74
Usefulness	0.00	10.00	7.29	2.01	–
Satisfaction	1.00	10.00	7.81	1.48	–
Frequency of Use	0.00	4.00	2.13	0.85	–
Future intention to use	1.00	7.00	4.89	1.19	0.89

Note. *M* = mean; *SD* = standard deviation; α = Cronbach's α; ω = McDonald's ω; AI = Artificial intelligence. *Usefulness*, *Satisfaction*, and *Frequency of Use* were assessed using single-item measures; therefore, internal consistency indices (McDonald's ω) were not applicable.

Regarding gender differences, women reported significantly higher levels of AI-related anxiety than men, with a moderate effect size, whereas men reported slightly higher facilitating conditions, with a small effect size. No statistically meaningful gender differences were observed for perceived risk, positive attitudes toward AI, acceptance and predisposition, prior experience, perceived usefulness, satisfaction, frequency of use, or future intention to use (Table S2).

Table S2: Descriptive statistics and differences by gender

Variable	**Men** (n = 57)		**Women** (n = 301)				
	**M**	**SD**	**M**	**SD**	***t (df)***	***p***	**Hedges' g**
AI Anxiety	82.14	20.18	93.43	20.60	−3.86 (79.68)	$< 0.001^{***}$	−0.55
Perceived Risk	4.26	1.09	4.26	0.95	0.03 (72.91)	0.974	0.00
Positive Attitudes	40.25	7.09	39.17	6.51	1.07 (75.00)	0.289	0.16
Facilitating Conditions	5.29	0.74	5.07	0.82	2.03 (83.99)	0.045^{*}	0.27
Acceptance & Predisposition	4.70	0.74	4.53	0.76	1.62 (79.65)	0.110	0.23
Prior Experience	2.35	0.77	2.55	0.71	−1.82 (74.65)	0.073	−0.28
Usefulness	7.51	1.92	7.25	2.03	0.89 (78.53)	0.375	0.13
Satisfaction	7.73	1.38	7.83	1.50	−0.49 (80.00)	0.625	−0.07
Frequency of Use	2.13	0.79	2.13	0.86	−0.06 (79.98)	0.955	0.00
Future intention to use	4.88	1.29	4.89	1.17	−0.03 (74.39)	0.973	−0.01

Note. *M* = mean; SD = standard deviation. Group differences were examined using Welch's independent-samples *t* tests; degrees of freedom (df) are reported in parentheses. Effect sizes are reported as Hedges' *g*. $^{*}p < .05$, $^{**}p < .01$, $^{***}p < .001$.

Differences by stage of training indicated that master's students reported more positive attitudes toward AI, whereas first-year undergraduate students reported higher facilitating conditions. Effect sizes ranged from small-to-moderate for positive attitudes to moderate-to-large for facilitating conditions. No additional differences were observed across training stages for the remaining variables (Table S3).

Table S3: Descriptive statistics and differences by stage of training

	**1st year** (n = 96)		**Master's** (n = 47)				
**Variable**	**M**	**SD**	**M**	**SD**	***t (df)***	***p***	**Hedges' g**
AI Anxiety	93.56	21.37	90.57	20.34	0.81 (95.61)	0.419	0.14
Perceived Risk	4.33	0.92	4.35	0.79	−0.14 (104.93)	0.887	−0.02
Positive Attitudes	38.07	6.20	40.36	5.66	−2.20 (99.33)	0.030^{*}	−0.38
Facilitating Conditions	5.28	0.76	4.78	0.81	3.57 (86.69)	0.001^{**}	0.67
Acceptance & Predisposition	4.56	0.78	4.60	0.58	−0.36 (118.27)	0.718	−0.06
Prior Experience	2.64	0.74	2.41	0.69	1.82 (97.77)	0.072	0.32
Usefulness	7.01	2.23	7.13	1.83	−0.35 (103.86)	0.731	−0.06
Satisfaction	7.78	1.55	8.09	1.26	−1.26 (104.79)	0.211	−0.22
Frequency of Use	2.24	0.85	2.02	0.75	1.55 (96.29)	0.125	0.28
Future intention to use	5.06	1.09	4.96	0.95	0.55 (103.76)	0.581	0.10

Note. M = mean; SD = standard deviation. Group differences were examined using Welch's independent-samples t tests; degrees of freedom (df) are reported in parentheses. Effect sizes are reported as Hedges' g. $^{*}p < .05$, $^{**}p < .01$, $^{***}p < .001$.

Finally, differences emerged as a function of frequency of AI use. Participants reporting high frequency of use showed more positive attitudes toward AI, greater facilitating conditions, higher acceptance and predisposition, greater prior experience, higher perceived usefulness, greater satisfaction, and stronger future intention to use, compared with low-frequency users. Effect sizes ranged from small-to-moderate to large, with the largest effects observed for prior experience and perceived usefulness. No differences were observed between frequency-of-use groups for perceived risk or AI-related anxiety (Table S4).

Table S4: Descriptive statistics and differences by frequency of AI use

	Low frequency (n = 236)		High frequency (n = 109)				
**Variable**	**M**	**SD**	**M**	**SD**	***t (df)***	***p***	**Hedges' g**
AI Anxiety	92.33	21.42	90.17	20.00	0.91 (223.84)	0.363	0.10
Perceived Risk	4.26	0.96	4.24	0.98	0.19 (206.95)	0.848	0.02
Positive Attitudes	38.62	6.16	41.55	6.47	−3.97 (201.04)	$< 0.001^{***}$	−0.47
Facilitating Conditions	4.97	0.80	5.43	0.73	−5.28 (227.36)	$< 0.001^{***}$	−0.60
Acceptance & Predisposition	4.42	0.70	4.89	0.74	−5.56 (198.46)	$< 0.001^{***}$	−0.66
Prior Experience	2.38	0.54	3.02	0.62	−9.35 (186.30)	$< 0.001^{***}$	−1.11
Usefulness	6.71	2.01	8.55	1.36	−9.97 (297.25)	$< 0.001^{***}$	−1.02
Satisfaction	7.49	1.49	8.49	1.20	−6.62 (256.79)	$< 0.001^{***}$	−0.74
Future intention to use	4.65	1.05	5.58	1.04	−7.62 (211.73)	$< 0.001^{***}$	−0.88

Note. M = mean; SD = standard deviation. Group differences were examined using Welch's independent-samples t tests; degrees of freedom (df) are reported in parentheses. Effect sizes are reported as Hedges' g. $^{*}p < .05$, $^{**}p < .01$, $^{***}p < .001$.

2 Sequential Regression Analyses

Each regression targeted a specific dependent variable corresponding to a distinct stage of the adoption process, allowing the identification of the most influential predictors at each step: AI-related anxiety, positive attitudes toward AI, perceived facilitating conditions, acceptance and predisposition toward AI, prior AI experience, frequency of use, and future intention to use AI technologies. All regression models were estimated using the lm() function from the base stats R package [1].

2.1 Identification of predictors within the AI adoption process

The first regression model examined predictors of AI-related anxiety. Results indicated that dispositional and personality-related factors played a prominent role. Higher levels of resistance to change (β = .21) and conspiratorial thinking (β = .19) emerged as the strongest positive predictors, followed by extraversion (β = .11). Emotional stability showed a negative association (β = $-$.12), indicating that lower emotional stability was linked to higher AI anxiety. Gender also showed a modest but significant effect (β = .14). Together, these predictors accounted for a meaningful proportion of variance in AI anxiety (adjusted R^2 = .19).

The second regression focused on positive attitudes toward AI. In this model, affective predictors clearly outweigh personality traits. Perceived risk (β = $-$.32) and AI anxiety (β = $-$.27) emerged as the dominant negative predictors, indicating that higher perceived threat and fear were associated with less favorable attitudes. Agreeableness showed a smaller negative effect (β = $-$.10). The model explained a moderate amount of variance in positive attitudes toward AI (adjusted R^2 = .22).

Subsequently, facilitating conditions were examined as the outcome. Positive attitudes toward AI showed the strongest positive association (β = .32), followed by openness to experience (β = .16) and resistance to change (β = .14). AI anxiety was negatively related to facilitating conditions (β = $-$.15), suggesting that higher fear was associated with less favorable perceptions of support and ease of use. This model accounted for a modest but meaningful proportion of variance (adjusted R^2 = .16).

The next regression examined acceptance and predisposition toward AI. This model showed a substantial increase in explanatory power (adjusted R^2 = .50). Facilitating conditions (β = .37) and positive attitudes toward AI (β = .44) emerged as the most influential predictors. Perceived risk showed a strong negative association (β = $-$.20), while AI anxiety (β = .13), resistance to change (β = .09), and age (β = .10) contributed smaller but significant effects.

The fifth regression focused on prior experience with AI technologies. Acceptance and predis-

position emerged as the strongest predictor (β = .37), followed by positive attitudes toward AI (β = .22) and perceived risk (β = .15). Extraversion (β = .12), gender (β = .14), and age ($\beta = -.12$) also contributed significantly. These findings suggest that experiential engagement with AI reflects both attitudinal readiness and individual characteristics (adjusted R^2 = .26).

Finally, future intention to use AI was examined as the ultimate outcome. Acceptance and predisposition showed a dominant effect (β = .60), followed by frequency of use (β = .20) and prior experience (β = .11). This model accounted for a substantial proportion of variance in future intention to use AI (adjusted R^2 = .60), underscoring the central role of attitudinal engagement and hands-on experience in shaping future adoption intentions. The relative importance of predictors across all blocks is illustrated in Figure S1. Complete regression outputs for each block are reported in Tables S5–S11.

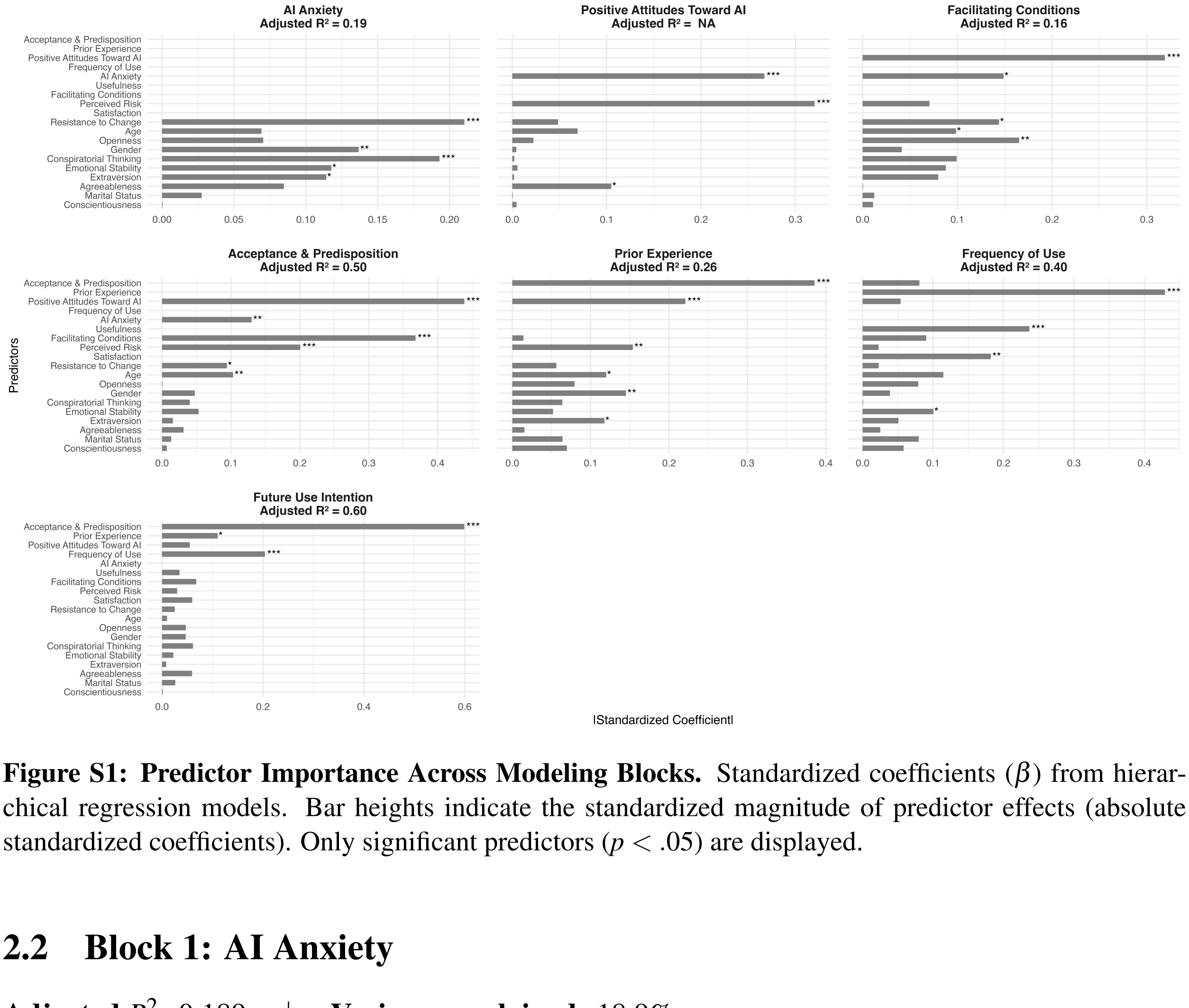

Figure S1: Predictor Importance Across Modeling Blocks. Standardized coefficients (β) from hierarchical regression models. Bar heights indicate the standardized magnitude of predictor effects (absolute standardized coefficients). Only significant predictors ($p < .05$) are displayed.

2.2 Block 1: AI Anxiety

Adjusted R^2: 0.189 | **Variance explained:** 18.9%

Table S5: Complete Regression Output for Block 1: AI Anxiety (Standardized Variables)

Predictor	Estimate	SE	t	p
Intercept	−0.007	0.048	−0.15	0.880
Age	−0.069	0.049	−1.41	0.159
Gender	0.137	0.049	2.79	0.005**
Marital Status	0.028	0.048	0.57	0.567
Extraversion	0.114	0.051	2.24	0.026*
Agreeableness	0.085	0.048	1.77	0.078
Conscientiousness	−0.000	0.050	−0.01	0.993
Emotional Stability	−0.118	0.056	−2.09	0.037*
Openness	−0.070	0.051	−1.39	0.165
Resistance to Change	0.210	0.056	3.74	< 0.001***
Conspiratorial Thinking	0.193	0.049	3.95	< 0.001***

*$p < .05$, **$p < .01$, ***$p < .001$

2.3 Block 2: Positive Attitudes Toward AI

Adjusted R^2: 0.225 | **Variance explained:** 22.5%

Table S6: Complete Regression Output for Block 2: Positive Attitudes Toward AI

Predictor	Estimate	SE	t	p
Intercept	−0.002	0.047	−0.04	0.972
Age	0.069	0.048	1.44	0.151
Gender	−0.004	0.049	−0.09	0.930
Marital Status	0.000	0.047	0.01	0.992
Extraversion	0.002	0.051	0.04	0.971
Agreeableness	−0.105	0.047	−2.21	0.028*
Conscientiousness	0.004	0.049	0.09	0.928
Emotional Stability	−0.005	0.056	−0.10	0.922
Openness	0.022	0.050	0.45	0.653
Resistance to Change	0.048	0.056	0.86	0.390
Conspiratorial Thinking	0.002	0.049	0.04	0.965

Continued on next page

Table S6 – Continued from previous page

Predictor	Estimate	SE	t	p
Perceived Risk	−0.320	0.052	−6.13	< 0.001***
AI Anxiety	−0.267	0.056	−4.73	< 0.001***

$^{*}p < .05$, $^{**}p < .01$, $^{***}p < .001$

2.4 Block 3: Facilitating Conditions

Adjusted R^2: 0.160 | **Variance explained:** 16.0%

Table S7: Complete Regression Output for Block 3: Facilitating Conditions

Predictor	Estimate	SE	t	p
Intercept	0.005	0.048	0.10	0.917
Age	−0.099	0.050	−1.97	0.050*
Gender	−0.041	0.051	−0.82	0.413
Marital Status	−0.012	0.049	−0.25	0.802
Extraversion	−0.080	0.053	−1.50	0.134
Agreeableness	0.001	0.050	0.01	0.992
Conscientiousness	0.011	0.051	0.22	0.829
Emotional Stability	0.088	0.058	1.52	0.130
Openness	0.165	0.052	3.21	0.001**
Resistance to Change	0.144	0.058	2.46	0.014*
Conspiratorial Thinking	0.099	0.051	1.95	0.053
Perceived Risk	0.071	0.057	1.24	0.217
AI Anxiety	−0.149	0.060	−2.46	0.014*
Attitudes Toward AI	0.319	0.056	5.69	< 0.001***

$^{*}p < .05$, $^{**}p < .01$, $^{***}p < 0.001$

2.5 Block 4: Acceptance & Predisposition

Adjusted R^2: 0.497 | **Variance explained:** 49.7%

Table S8: Complete Regression Output for Block 4: Acceptance & Predisposition

Predictor	Estimate	SE	t	p
Intercept	−0.004	0.038	−0.10	0.925
Facilitating Conditions	0.368	0.042	8.76	$< 0.001^{***}$
Age	0.103	0.039	2.63	0.009^{**}
Gender	−0.048	0.039	−1.21	0.226
Marital Status	0.013	0.038	0.35	0.729
Extraversion	0.016	0.041	0.38	0.704
Agreeableness	−0.031	0.039	−0.81	0.418
Conscientiousness	−0.007	0.040	−0.18	0.859
Emotional Stability	0.053	0.045	1.18	0.240
Openness	0.001	0.041	0.02	0.985
Resistance to Change	0.094	0.046	2.06	0.041^{*}
Conspiratorial Thinking	0.040	0.040	1.01	0.312
Perceived Risk	−0.201	0.044	−4.51	$< 0.001^{***}$
AI Anxiety	0.130	0.047	2.75	0.006^{**}
Attitudes Toward AI	0.439	0.046	9.63	$< 0.001^{***}$

$^{*}p < .05$, $^{**}p < .01$, $^{***}p < .001$

2.6 Block 5: Prior Experience

Adjusted R^2: 0.258 | **Variance explained:** 25.8%

Table S9: Complete Regression Output for Block 5: Prior Experience

Predictor	Estimate	SE	t	p
Intercept	0.001	0.046	0.01	0.990
Age	−0.120	0.048	−2.50	0.013^{*}
Gender	0.145	0.047	3.07	0.002^{**}
Marital Status	−0.064	0.046	−1.38	0.167
Extraversion	0.117	0.050	2.36	0.019^{*}
Agreeableness	0.016	0.047	0.33	0.739
Conscientiousness	−0.070	0.048	−1.44	0.150

Continued on next page

Table S9 – Continued from previous page

Predictor	Estimate	SE	t	p
Emotional Stability	0.052	0.055	0.95	0.344
Openness	−0.079	0.049	−1.61	0.109
Resistance to Change	0.056	0.055	1.02	0.307
Conspiratorial Thinking	0.064	0.048	1.33	0.184
Perceived Risk	0.154	0.053	2.88	0.004**
Attitudes Toward AI	0.221	0.061	3.64	< 0.001***
Facilitating Conditions	−0.014	0.056	−0.26	0.799
Acceptance & Predisposition	0.386	0.065	5.92	< 0.001***

*$p < .05$, **$p < .01$, ***$p < .001$

2.7 Block 6: Frequency of Use

Adjusted R^2: 0.400 | **Variance explained:** 40.0%

Table S10: Complete Regression Output for Block 6: Frequency of Use

Predictor	Estimate	SE	t	p
Intercept	−0.035	0.042	−0.82	0.411
Age	−0.115	0.062	−1.85	0.065
Gender	−0.039	0.044	−0.88	0.380
Marital Status	−0.080	0.043	−1.87	0.063
Extraversion	−0.051	0.047	−1.07	0.283
Agreeableness	−0.025	0.043	−0.59	0.557
Conscientiousness	0.058	0.044	1.32	0.187
Emotional Stability	−0.101	0.051	−1.97	0.049*
Openness	−0.079	0.046	−1.73	0.085
Resistance to Change	−0.023	0.051	−0.44	0.654
Conspiratorial Thinking	0.001	0.045	0.02	0.988
Perceived Risk	0.023	0.049	0.46	0.647
Attitudes Toward AI	0.054	0.057	0.94	0.346
Facilitating Conditions	0.090	0.052	1.75	0.081

Continued on next page

Table S10 – Continued from previous page

**Predictor**	**Estimate**	**SE**	***t***	***p***
Acceptance & Predisposition	−0.081	0.066	−1.22	0.223
Prior Experience Composite	0.429	0.056	7.68	$< 0.001^{***}$
Satisfaction	0.182	0.055	3.31	0.001^{**}
Usefulness	0.237	0.057	4.18	$< 0.001^{***}$

$^{*}p < .05$, $^{**}p < .01$, $^{***}p < .001$

2.8 Block 7: Future Intention to Use

Adjusted R^2: 0.603 | **Variance explained:** 60.3%

Table S11: Complete Regression Output for Block 7: Future Intention to Use

**Predictor**	**Estimate**	**SE**	***t***	***p***
Intercept	0.018	0.033	0.54	0.589
Age	0.010	0.049	0.20	0.839
Gender	0.047	0.035	1.36	0.176
Marital Status	0.026	0.034	0.78	0.436
Extraversion	0.008	0.037	0.22	0.826
Agreeableness	−0.060	0.033	−1.78	0.076
Conscientiousness	0.001	0.035	0.04	0.967
Emotional Stability	0.023	0.040	0.56	0.575
Openness	−0.047	0.036	−1.31	0.191
Resistance to Change	−0.025	0.040	−0.63	0.528
Conspiratorial Thinking	−0.061	0.035	−1.76	0.080
Perceived Risk	0.030	0.039	0.77	0.440
Attitudes Toward AI	0.055	0.045	1.23	0.221
Facilitating Conditions	0.068	0.041	1.67	0.096
Acceptance & Predisposition	0.599	0.052	11.54	$< 0.001^{***}$
Prior Experience Composite	0.110	0.048	2.32	0.021^{*}
Satisfaction	−0.060	0.044	−1.37	0.173
Usefulness	−0.035	0.046	−0.76	0.446

Continued on next page

Table S11 – Continued from previous page

**Predictor**	**Estimate**	**SE**	***t***	***p***
Frequency of Use	0.204	0.043	4.70	$< 0.001^{***}$

$^{*}p < .05$, $^{**}p < .01$, $^{***}p < .001$

3 Structural Equation Modeling Results

3.1 Direct, Indirect, and Total Effects

The following table presents the complete set of path coefficients from the PLS-SEM analysis, including direct effects, indirect (mediated) effects, total effects, and 95% confidence intervals for the total effects based on 5,000 bootstrap resamples.

Table S12: Direct, Indirect, and Total Effects from PLS-SEM Analysis (5,000 Bootstrap Resamples)

**Path**	**Direct β**	**Indirect β**	**Total β**	**95% CI (Total)**
Predisposing factors				
Extraversion → Fears	0.13	0.00	0.13	[0.03, 0.23]
Conspiratorial Thinking → Fears	0.19	0.00	0.19	[0.09, 0.29]
Resistance to Change → Fears	0.28	0.00	0.28	[0.18, 0.38]
Gender → Fears	0.16	0.00	0.16	[0.07, 0.25]
Precipitating factors				
Fears → Perceived Risk	0.37	0.00	0.37	[0.27, 0.47]
Fears → Positive Attitudes	−0.28	−0.11	−0.39	[−0.48, −0.30]
Perceived Risk → Positive Attitudes	−0.30	0.00	−0.30	[−0.42, −0.18]
Perceived Risk → Facilitating Conditions	0.06	−0.11	−0.05	[−0.17, 0.07]
Perceived Risk → AI Acceptance & Predisposition	−0.14	−0.15	−0.29	[−0.40, −0.18]
Positive Attitudes → Facilitating Conditions	0.36	0.00	0.36	[0.22, 0.49]
Positive Attitudes → AI Acceptance & Predisposition	0.42	0.13	0.56	[0.46, 0.65]
Facilitating Conditions → AI Acceptance & Predisposition	0.37	0.00	0.37	[0.29, 0.46]

Continued on next page

Table S12 – Continued from previous page

Path	Direct β	Indirect β	Total β	95% CI (Total)
Maintaining factors				
AI Acceptance & Predisposition → Prior Experience	0.52	0.00	0.52	[0.45, 0.59]
AI Acceptance & Predisposition → Satisfaction	0.35	0.09	0.44	[0.36, 0.53]
AI Acceptance & Predisposition → Usefulness	0.42	0.12	0.54	[0.45, 0.63]
AI Acceptance & Predisposition → Future intention to use	0.67	0.10	0.76	[0.71, 0.81]
Prior Experience → Satisfaction	0.18	0.00	0.18	[0.08, 0.28]
Prior Experience → Usefulness	0.24	0.00	0.24	[0.14, 0.34]
Prior Experience → Usage Frequency	0.47	0.07	0.54	[0.45, 0.62]
Prior Experience → Future intention to use	0.00	0.13	0.13	[0.08, 0.18]
Satisfaction → Usage Frequency	0.13	0.00	0.13	[0.02, 0.24]
Satisfaction → Future intention to use	0.00	0.03	0.03	[0.01, 0.06]
Usefulness → Usage Frequency	0.19	0.00	0.19	[0.10, 0.29]
Usefulness → Future intention to use	0.00	0.05	0.05	[0.02, 0.07]
Usage Frequency → Future intention to use	0.23	0.00	0.23	[0.16, 0.31]

Note. Direct effects represent the unmediated influence of one construct on another. Indirect effects represent the mediated influence through intervening variables. Total effects are the sum of direct and indirect effects. Confidence intervals are based on percentile bootstrap methods with 5,000 resamples. Paths with direct $\beta = 0.00$ indicate fully mediated relationships where the effect is entirely indirect.

3.2 Effect Size Analysis

To evaluate the practical significance of the structural relationships identified in the PLS-SEM model, we calculated Cohen's f^2 effect sizes for each predictor-outcome path. Effect sizes provide a standardized measure of the magnitude of each predictor's contribution to the explained variance in its target construct, independent of sample size considerations. Following Cohen's (1988) conventional benchmarks, f^2 values of 0.02, 0.15, and 0.35 represent small, medium, and large effects, respectively.

Table S13 presents the effect sizes organized by process stage (predisposing, precipitating, and maintaining factors), reflecting the empirical ordering of constructs in our theoretical model. The results reveal several noteworthy patterns. First, among predisposing factors, Resistance to Change exhibited the strongest effect on AI Anxiety (f^2 = 0.09), approaching a medium effect size. Second, in the precipitating stage, Attitudes toward AI demonstrated substantial effects on both Facilitating Conditions (f^2 = 0.12) and AI Acceptance (f^2 = 0.27), with the latter showing a medium-to-large effect. Third, the maintaining factors revealed that AI Acceptance exerted the largest effect on Prior Experience (f^2 = 0.37) and an exceptionally large effect on Future intention to use (f^2 = 1.00), underscoring its central role in predicting sustained engagement with AI technologies.

Table S13: Cohen's f^2 Effect Size Estimates for the PLS-SEM Structural Model

**Predictor**	**Outcome**	**Process Stage**	f^2	**Magnitude**
Predisposing factors				
Extraversion	AI Anxiety	Predisposing	0.02	Small
Conspiratorial Thinking	AI Anxiety	Predisposing	0.04	Small
Resistance to Change	AI Anxiety	Predisposing	0.09	Small–Medium
Gender	AI Anxiety	Predisposing	0.03	Small
Precipitating factors				
AI Anxiety	Perceived Risk	Precipitating	0.16	Medium
AI Anxiety	Attitudes	Precipitating	0.09	Small–Medium
Perceived Risk	Attitudes	Precipitating	0.10	Small–Medium
Attitudes	Facilitating Conditions	Precipitating	0.12	Medium
Attitudes	AI Acceptance	Precipitating	0.27	Medium–Large
Facilitating Conditions	AI Acceptance	Precipitating	0.25	Medium–Large

Continued on next page

Table S13 – Continued from previous page

**Predictor**	**Outcome**	**Process Stage**	f^2	**Magnitude**
Maintaining factors				
AI Acceptance	Prior Experience	Maintaining	0.37	Large
AI Acceptance	Satisfaction	Maintaining	0.12	Medium
AI Acceptance	Perceived Utility	Maintaining	0.19	Medium
Prior Experience	Usage Frequency	Maintaining	0.30	Medium
Satisfaction	Usage Frequency	Maintaining	0.02	Small
Perceived Utility	Usage Frequency	Maintaining	0.04	Small
AI Acceptance	Future intention to use	Maintaining	1.00	Large
Usage Frequency	Future intention to use	Maintaining	0.12	Medium

Note. Effect size interpretation follows Cohen (1988): small (f^2 = .02), medium (f^2 = .15), and large (f^2 = .35). Process stages reflect the empirical ordering of constructs in the structural model.

References

[1] R Core Team. R: A Language and Environment for Statistical Computing. Vienna, Austria; 2025. Available from: https://www.R-project.org/.
